# The Origin of Pathological Tendencies

**Published:** 1895-05

**Authors:** James B Hodgkin


// THE DENTAL REGISTER.

VOL. XLIX.] MAY, 1895. [No. 5.

### COMMUNICATIONS.

### The Origin of Pathological Tendencies.

BY JAMES B HODGKIN, D.D.S.

We shall never know the secret of life: for to know this will be to be as the gods—the life-makers. The history of the primordial germ will elude our keenest analysis, and neither scalpel or microscope will ever tell the secret of the original concept. Still, the desire to know, the restless wish to get to the fruit of this tree of knowledge will not be thwarted by difficulty, or turned aside by obscurity; for man still hopes to find that which makes him what he is.

Pathos, pain, sickness, organic weakness—how shall we inquire into the hidden nature of these, how study out the origin of the why one organ breaks down sooner than another; why this man's lungs go to pieces and that one's nervous system; why this woman has a lifelong bowel trouble and that one hysteria. We are, indeed, fond of saying that these are born with such and such tendencies, but beyond that and deeper than that lies the *why* it should be so. It is the province of this paper to look, so far as the writer's feeble powers may, into the causes that seem to go a step further into the case than simply the phrase "inheritance" implies. In other words, it is a question a step further than inheritance—how, or in what way, does the inheritance come? We can never hope to get all of this, never expect to fathom the mystery of nature, whereby she fashions cells, and from impressions so vague and occult as not to betray their presence save by their results the whole work is biased, leaning this way or that, is predisposed to this or that sort of ultimate breakdown; to know all this is such knowledge as we can only hope for, not expect.

Certainly we are a long way off from such knowledge as yet. Are we nearer to it, than we are, as to why man grows to be six feet?

One thing is certain, there is, of all nature's constancies, nothing so persistent as type. Moving within certain lines and developing within certain channels, nature is, with but slight deviations, one. The man of to-day is wonderfully like his ancestor the savage; so like that we are puzzled to know if the skull we dig up was buried one hundred or one thousand years ago, and mentally this is more true than we are willing to confess. Where is the poet since Homer? or the lawgiver since Moses? There is no architect since he who built Thebes; no profounder thinker than Plato. Reproduction, not creation, is man's mission, and reproduction within certain well-defined limits.

But in reproduction man has been allowed, if I may so speak, to reproduce not only his individuality, physiologically, but in some degree his abnormalities and weaknesses. All, however, within certain limits. Over all and above all the slow and unconsciously moving hand of evolution fashions him into something better? or worse? who shall say?

Why one man should be so constituted physically as to be predisposed to pneumonia, another to heart or kidney, or nervous trouble is, on the face of things, impossible to answer. We flippantly say it is inherited. But *why* inherited? The type persists: six feet high, and perfect, so far as functions go; what is that subtle and intangible thing we call hereditary predisposition? How far can we go in analyzing and studying this subtility?

With the two forces operating—one strictly enforcing the type, and the other causing within certain limits, physiological deviations from that type, there seems to me to be a *third* or possibly a modification of the second force. Pathological conditions, or rather the tendency to pathological conditions, seem as strictly inheritable as physiological ones. That constitutional bias by virtue of which certain diseases are likely to be developed seems to be as much an inheritance as symmetry of form. So we find, as hinted above, that almost every constitution has its predispositions, and these are as distinctive as the color of the eye or the form of the hand, the shade of the hair, etc. One family is predisposed to consumption, another to paralysis, and so on to the end of a long chapter. And even a supernumerary finger is as inheritable as a bad temper. We say of a certain man: "He goes too fast, walks, works too rapidly," and we charge his hurry with making him nervous. Really it is his nervous force that makes him go—and this he inherits.

Granted then that constitutional tendencies are inheritable and inherited, the question arises, in what way? We read in the 30th chapter of Genesis, that Jacob, whom Shakespeare makes Shylock to call "the crafty," placed before the breeding ewes certain colored rods, which these animals, seeing, would be maternally impressed by, and conceiving under these circumstances, bore offspring of certain colors. The impression here made is at the moment of conception, and it is to this phase of life, or life's beginning, that I wish to call attention. For of those obscure fœtal changes, resulting in monstrosities, which occur in intra-uterine life, of which so much is made by the ignorant, and so little made by those that closely observe, I have only to quote the saying of William Hunter, that in a life spent in a lying-in hospital he had never seen a coincidence—that is, had never seen a case in which the birth-marks where they existed, corresponded with the mother's expectations.

A widow, remarrying, will bear children like her first husband; a female, of any species, receives from her first sexual contact an impress that persists through many successive pregnancies. Thus a female dog will have successive litters of puppies like the father of her first litter, although there be no resemblance between him and the father of her second, third or fourth. Some mysterious stamp is made on the nervous system by this first contact, that modifies in an obscure way, many succeeding pregnancies.

Taking these two forces then, ,—that of the persistence of type: that of the differentiation of type within certain limits, and that third, or modification of the second force—the inheritance of pathological tendencies, and grouping these, we have the sum of what constitutes our "mold."

The question at the bottom is—at what time or in what way are these semi-pathological impressions, which we are obliged to recognize as existing, made on the constitution? Is it the "nine moons that go to the making of a man," as Tennyson has it; is it from accidental or incidenial impressions made on the fœtus in utero through the nervous system of the mother, as some are fond of imagining? I cannot think this is true. Nature does not thus work. I will illustrate what I mean by what we see oftener than we ought:

We have the picture, sometimes in the courts, sometimes in private life, of the seduced and betrayed woman abandoned to her fate by her betrayer. He refuses, after gratifying his lust, to make the amends for his crime he might, and leaves her to pine in solitude until she comes in the course of months to the maternal period. Imagine, if you can, what a torrent of feelings possess her. Now rage, now desperation, now despair, now hope. I cannot imagine a more troubled sea of unrest than the mind and soul of such a woman. Shame, fury, revenge, love, despair—a commingling of all that would make a turmoil of the soul, a hell on earth possesses her until the somber and to her disgraceful end. Suicide, murder, entreaty, all struggle for place in her perturbed soul. Did all these make their impress on the mind and character of her unborn son, what a monster he must be. Yet I am assured that some of the world's most illustrious men have been bastards, and born under circumstances such as I have depicted. I was told not long ago that on the bench of the court of appeals of one of the oldest and most honored of our States have sat at one time three bastards out of a total of four comprising the court; great and noble men, but legally fatherless.

It comes to me with more and more of increasing conviction that we are to look to the moment of conception for pathological tendencies no less than physiological stamp. When spermatozoa and ovum come together and the edict goes forth that "a man child is conceived," I cannot but think that he takes his ideal shape then and there. If he is to be tall or short, to have blue eyes or black, have his mother's characteristics or his father's—that all this is the result of the culminating stamp of concept; and that as Minerva sprang full-fledged from the brain of Jove, so the created man is created with all his peculiarities and idiosyncracies, his constitutional bias, then and there.

Hence we see in this light of the subject that a child has teeth the form of the mother and the quality of the father, or the reverse, or the jaws of one and the teeth of the other. But I need pursue this thought.

Such a theory relieves us from the necessity of believing that man is the creature of circumstances, and of the numberless accidents and impressions of intra-uterine life; it shows beyond doubt that in this way, and this way only, can the type be preserved. It relieves us from the necessity of accepting the doctrine that teeth can be starved in an otherwise well-nurtured body; and that, save in the case of accidental interference with nutrition, as in the case of an eruptive fever, or the pitted and semi-lunar markings of syphilitic inheritance, they are molded in the form and after the style of their ancestors.

It has been years since I first began to feel that we, as creatures could not be the helpless victims of circumstances, the sport of environment, the makeshift of accident. That man is the creature of his environments is a truism, broad and general, but too broad to be more than of very general application. But he is not the defenceless and helpless thing that such a doctrine would make him. Not only man but even the inferior animals mold circumstances and take advantage of ebb and flow to gain the desired haven. And we have only to look about us to see that man as much creates the world in which he dwells as he is created by it. His nature clings to type with great tenacity, and I do fail to see that environments have much modified that great fact.

If this paper has any "central idea," it is the attempt to show that the creative stamp is greater than any environing influence, and that not by slow aggregations but by a full, complete and perfectly accomplished act is the creative concept made. The development of the individual "ego" is the result of that crowning inception, and not the result of emergencies and trivialities. In the light of this basal theory we see how semi-pathological conditions by heredity become constitutional, and transmit themselves by one swift act to their successors, and by the individuality of primal stamp become a part of the person. Weak teeth, weak eyes, weak lungs—any weak organ is transmitted, and as pathologists we have to battle with the weakness.

